# Distinguishing characteristics of *Staphylococcus schleiferi* and *Staphylococcus coagulans* of human and canine origin

**DOI:** 10.1371/journal.pone.0296850

**Published:** 2024-02-08

**Authors:** Alaa H. Sewid, Stephen A. Kania

**Affiliations:** 1 Department of Microbiology, Faculty of Veterinary Medicine, Zagazig University, Zagazig, Egypt; 2 Department of Biomedical and Diagnostic Sciences, University of Tennessee, Knoxville, Tennessee, United States of America; Rutgers Biomedical and Health Sciences, UNITED STATES

## Abstract

*Staphylococcus schleiferi* and *Staphylococcus coagulans* are opportunistic pathogens of animals and humans. They were previously classified as *Staphylococcus schleiferi subs*. *schleiferi* and *Staphylococcus schleiferi subs*. *coagulans*, respectively, and recently reclassified as separate species. *S*. *coagulans*, is frequently associated with dogs, whereas *S*. *schleiferi* is more commonly isolated from humans. Coagulase activity status is a defining characteristic of the otherwise closely related species. However, the use of coagulase tests originally developed to distinguish *S*. *aureus* from non-coagulase-producing staphylococci, for this purpose is questionable and the basis for their host preference has not been elucidated. In the current study, a putative *coa* gene was identified and correlated with coagulase activity measured using a chromogenic assay with human and bovine prothrombin (closely related to canine prothrombin). The results of the tests performed with human prothrombin showed greater reactivity of *S*. *coagulans* isolates from humans than isolates obtained from dogs with the same substrate. Our data suggest that unlike S. *coagulans* isolates from humans, isolates from dogs have more coagulase activity with bovine prothrombin (similar to canine prothrombin) than human prothrombin. Differences in *nuc* and 16s rRNA genes suggest a divergence in *S*. *coagulans* and *S*. *schleiferi*. Phenotypic and genotypic variation based on the number of IgG binding domains, and the numbers of tandem repeats in C-terminal fibronectin binding motifs was also found in protein A, and fibronectin-binding protein B respectively. This study identified a *coa* gene and associated phenotypic activity that differentiates *S*. *coagulans* and *S*. *schleiferi* and identified key phylogenetic and phenotypic differences between the species.

## Introduction

*Staphylococcus schleiferi* is considered a coagulase-negative human pathogen responsible for surgical site and wound infections, pediatric meningitis, endocarditis, osteomyelitis, and device-related bacteremia [1–7]. *Staphylococcus coagulans* is frequently isolated from dogs and cats as the second most common coagulase-positive species after *Staphylococcus pseudintermedius* [8–10] and is also occasionally associated with disease in humans [11]. *S*. *schleiferi* and *S*. *coagulans* were previously classified as *Staphylococcus schleiferi subs*. *schleiferi* and *Staphylococcus schleiferi subs*. *coagulans*, respectively, and reclassified based on core genome phylogeny complemented with genome-based indices [12, 13]. Coagulase activity is considered a major distinguishing characteristic of the two species [14]. This is often based on tube coagulase testing using prothrombin contained within rabbit plasma to detect free coagulase activity [15] and slide agglutination tests for bound coagulase (clumping factor) [16, 17] although the bound coagulase test is of questionable utility [9]. However, other sources of plasma and other testing protocols may be used [18] and the tests used in clinical laboratories were generally developed to identify *Staphylococcus aureus* rather than to distinguish *S*. *schleiferi* from *S*. *coagulans*. *S*. *aureus*-based coagulase testing likely underestimates the frequency of *S*. *coagulans* causing human infections due to the lower reactivity of *S*. *coagulans* compared to *S*. *aureus*. This is important because coagulase-negative staphylococci are thought to be of less clinical concern because they have fewer virulence factors than coagulase-positive members of the genus. A previous study showed that *S*. *coagulans* produces an extracellular protein similar to staphylocoagulase that can conformationally activate prothrombin and cleave fibrinogen to fibrin [15]. However, the protein responsible for this activity and its host preference is not well defined in *S*. *coagulans*. Our preliminary analysis revealed that only one *S*. *coagulans* gene contains domains associated with bacterial coagulase activity absent in species closely related to *S*. *schleiferi*. Studies with other bacterial species have shown a host related ability for coagulase to act on prothrombin from different mammalian sources [19]. For this reason, we sought to detect the coagulase gene as a useful way to differentiate *S*. *coagulans* from *S*. *schleiferi* and determine if there is a host preference for *S*. *coagulans* free coagulase associated with its source of isolation. In a study of coagulase-positive staphylococci, it was reported that *nuc* gene phylogeny was indistinguishable between *S*. *schleiferi* and *S*. *coagulans* [20]. We sought to examine whether the *nuc* gene might differ between the species based on their source of isolation.

The success of *S*. *schleiferi* in colonization and persistence at various sites of their hosts suggests a similarity of cell surface protein receptor MSCRAMMs (microbial surface components recognizing adhesive matrix molecules) to those of coagulase- positive staphylococci including *S*. *aureus* [21]. Members of this receptor family include several proteins associated with bound coagulase activity, that act directly on fibrinogen, including fibronectin-binding proteins A and B that bind fibronectin, fibrinogen, and elastin [22] and clumping factor proteins A and B that promote attachment to fibrinogen [23]. Staphylococcal protein A binds immunoglobulin at the cell surface, playing a similar role in protecting the bacteria from host defenses [24]. Our preliminary genetic analysis revealed the presence of several genes showing similarity with *S*. *aureus clfB*, *fnbpB*, and *spa* genes with bacterial proteins that bind to fibrinogen, fibronectin, and IgG.

The aims of this study were to identify the *S*. *coagulans* gene associated with coagulase activity, examine cell surface-associated virulence factors in *S*. *schleiferi* and *S*. *coagulans*, and to identify potential genotypic and phenotypic differences. We sought to relate these characteristics to the host source of isolation to identify potential bases for a selective advantage.

## Results

### 16S rRNA gene and *nuc* sequencing

**Fig 1:**
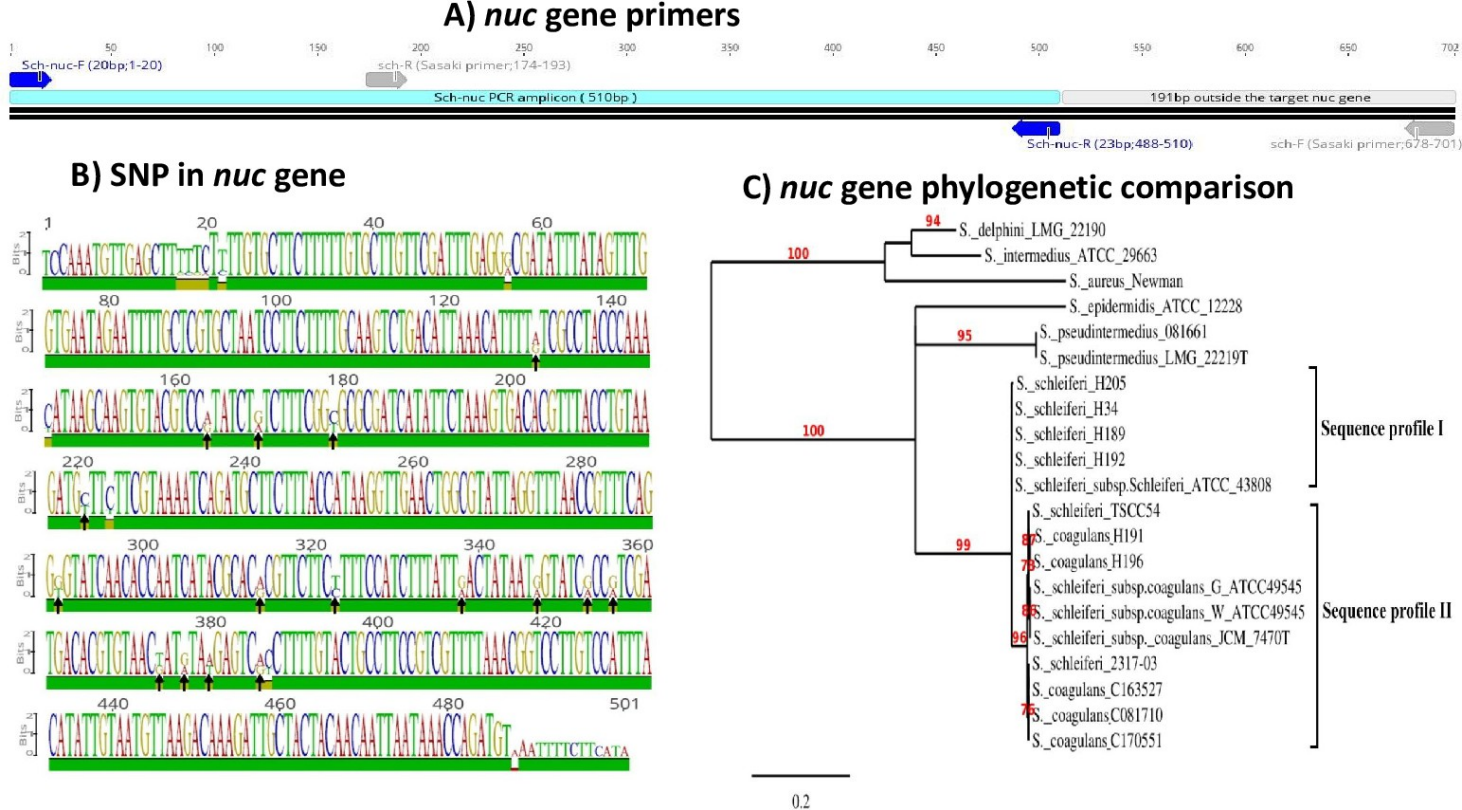
Thermonuclease gene phylogenetic comparison. Panel A) The binding site of sch-nuc primers used in this study compared with previously published primer pairs. Panel B) Sequence logos of the *S*. *schleiferi* and *S*. *coagulans* thermonuclease (*nuc*) genes. Sequence logos created by Geneious 2019.2.1 software show a 16 nucleotide difference between the two species. Panel C) Phylogenetic tree based on complete *nuc* sequences in staphylococci showing the relationships among *S*. *schleiferi* and *S*. *coagulans* of human (H) and canine (C) origin. The scale bar indicates the amount of sequence divergence over the length of the bar as a decimal percentage (0.2 equals 20%).Bootstrap probability is expressed as percentages indicated at diverging points of branches. Braces indicate the two *nuc* sequence profiles; sequence profile I is similar to the *S*. *schleiferi* and sequence profile II is similar to the *S*. *coagulans nuc* sequence. The following nuclease gene sequences were obtained from the GenBank database (accession numbers): *S*. *aureus subsp*. *aureus* strain Newman (CP023391.1), *S*. *epidermidis* strain ATCC 12228 (CP022247.1,), *S*. *intermedius* ATCC 29663(AB327165.1), *S*. *schleiferi subsp*. *coagulans* JCM 7470T(AB465334), *S*. *schleiferi* TSCC54 (AP014944.1), *S*. *schleiferi* 2317-03(CP010309.1), *S*. *delphini* LMG 22190 (AB327167.2), *S*. *pseudintermedius* 081661 (CP016073.1), and *S*. *pseudintermedius* LMG 22219T (AB327164).

*Nuc* and 16S rRNA gene sequencing were performed as part of the process to determine if *S*. *coagulans* and *S*. *schleiferi* isolates from dogs and humans were phylogenetically distinguishable. A consistent difference was found between the two species at 16 locations in the *nuc* gene (Fig 1 and S1 Fig**)** and a single nucleotide polymorphism near the 5’ end of the 16S rRNA gene (relevant section shown in S2 Fig) corresponding to the presence or absence of the *coa* gene (described below). Thus, within the samples tested, a single SNP distinguishes between *S*. *coagulans* and *S*. *schleiferi*.

### *coa* gene detection and measurement of coagulase activation of prothrombin

A PCR product was produced from all *S*. *schleiferi* of canine origin and 45.5% of human origin using the Sch-coa primer pair (Table 1). This included all 30 *S*. *coagulans* and 2 (H191 and H196) out of 20 *S*. *schleiferi* isolates. None of the non-*S*. *schleiferi* or *S*. *coagulans* reference strains (coagulase-positive members of the *S*. *intermedius* group) were amplified with this primer set and the *S*. *schleiferi* subsp. *schleiferi* (now *S*. *schleiferi*) ATCC 43808 control strain was negative. A *coa* PCR product was produced from coagulase-positive members of the *S*. *intermedius* group including *S*. *pseudintermedius*, *S*. *intermedius*, and *S*. *delphini* using a previously designed primer, pseud-coa, whereas none of the *S*. *schleiferi* and *S*. *coagulans* isolates produced a PCR product using this primer.

**Table 1:** List of primers used in this study.

Gene	Primer Sequence (5’ - 3’)	Size of PCR product (bp)	Strain, Binding site of the primer	Reference
sch- nuc (PCR1)	F-AATGGCTACAATGATAATCACTAA	526 bp		[20]
	R-CATATCTGTCTTTCGGCGCG			
sch- nuc (PCR2)	F-TTACGCTTCACTCCAAATGT	510 bp	*1360-13(1489825–1490334)	This study
	R-ATGAAGAAATTTACATCTGGTTT			
			*2142-05(1487772–1488281)	
			*5909-02(1554619–1555128)	
			*2317-03(1609088–1609597)	
			*TSCC54(1558594–1559103)	
16S rRNA	F-GCGGATCCTGCAGAGTTTGATCCTGGCTCAG	1500 bp		[32]
	R-GGCTCGACCGGGTTACCTTGTTACGACTT			
sch- coa	F-TTTGGCCATGGATGAAAAAGAAGTTAGTT	1500 bp	*1360-13(1024785–1026293)	This study
	R-TTTGGGGATCCTTGACCGTTATATGCTTTA		*2142-05(1024820–1026316)	
			*5909-02(1049907–1051403)	
			*2317-03(1089135–1090631)	
			*TSCC54(1091258–1092760)	
pse- coa	F-TTTGGCCATGGATGAAAAAGAAATTGCTT	1500 bp	*081661(2612742–2614241)	This study
			*NCTC11048(6187–7683)	
	R-TTTGGGGATCCTGACCGTTGTAAGCTTTAT			
			*8086(20325–21818)	
sch- clfB	F-ATGAAAAAATCGAAAAGACT	2589 bp	TSCC54(622001–624589)	This study
	R-CTATTGCTGATCTTTACGGCG			
fnbA	F-GGCCAAAATAGCGGTAACC	345 bp		[29]
	R-GCTTACTTTTGGAAGTGTATC			

**Fig 2:**
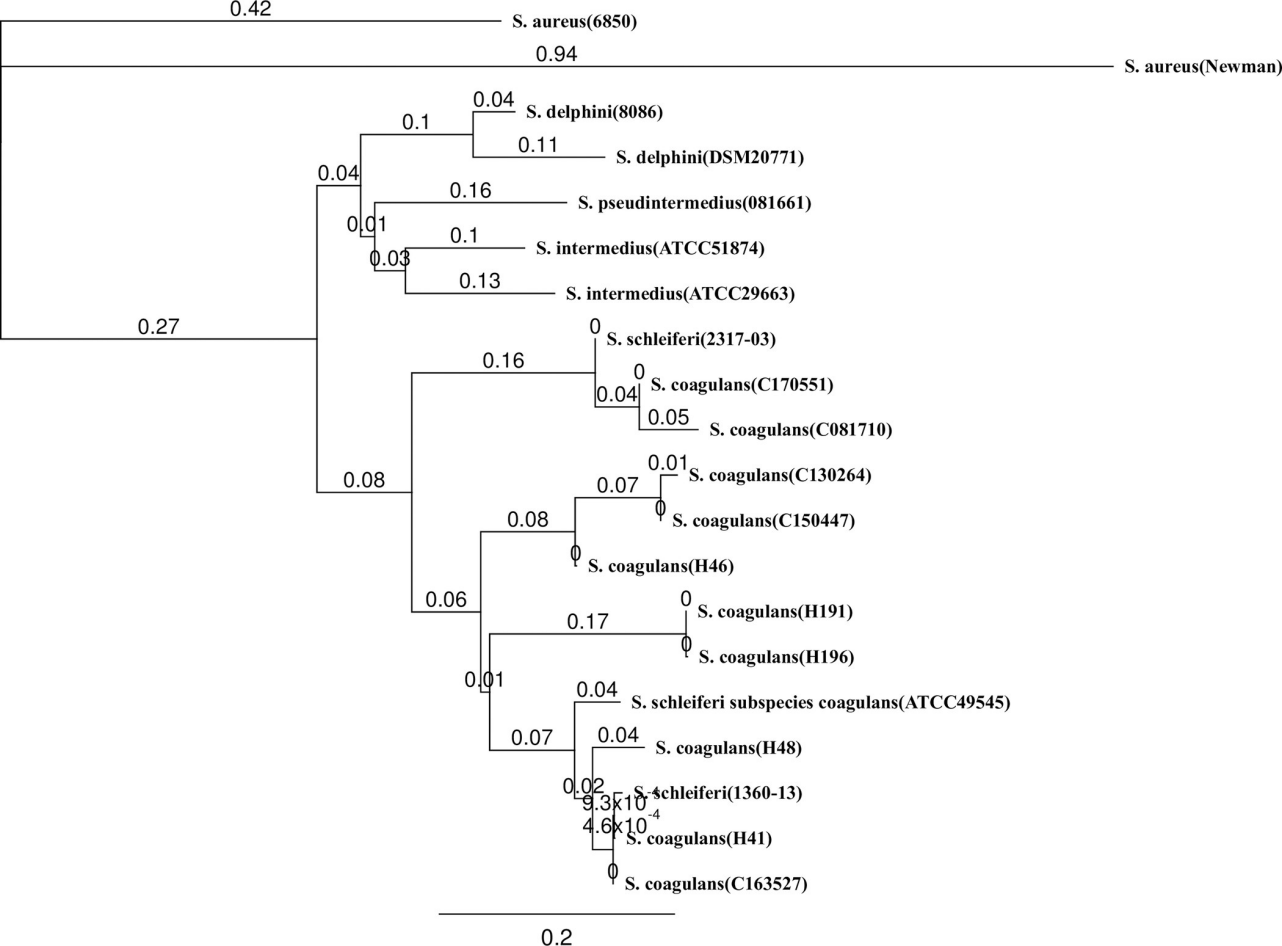
Coagulase gene phylogenetic comparison. Phylogenetic tree based on complete coagulase (coa) protein sequences showing the relationships among 19 species of the genus *Staphylococcus* including 13 *S*. *coagulans* of human (H) and canine (C) origin. The scale bar indicates the sequence divergence. Bootstrap probabilities are expressed as percentages and are shown at diverging points of branches. The following staphylocoagulase protein sequences were obtained from the GenBank database (accession numbers): *S*. *aureus* subsp. *aureus* strain Newman (WP_000744074), *S*. *intermedius* ATCC 29663(WP_019169028.1), *S*. *schleiferi* 1360-13(WP_050345467.1), *S*. *schleiferi* 2317-03(WP_050330609.1), *S*. *delphini* 8086(WP_019166910.1), and *S*. *pseudintermedius* 081661 (WP_037544060.1).

The results of DNA sequencing confirmed that *S*. *coagulans* isolates from both human and canine origin encode a protein that is similar in sequence and organization to coagulase proteins found in coagulase-positive members of the *S*. *intermedius* group, with sequence homologies of 68%, 66%, and 64% compared to *S*. *pseudintermedius*, *S*. *delphini* and *S*. *intermedius*, respectively (Fig 2). It differs from *S*. *aureus* Newman (26% identity) and *S*. *aureus* 6850 (41% identity) determined by BLAST analysis. The predicted protein has an N-terminal prothrombin binding site with 30% and 49% identity to the N-terminal prothrombin binding D1 and D2 domains of *S*. *aureus* strains Newman and 6850, respectively (S3 Fig), and C-terminal fibrinogen binding region, with 48% and 68% identity to the C-terminal fibrinogen binding regions of the same strains. The two *coa* PCR positive *S*. *schleiferi* isolates have identical deletions in their *coa* relative to the *coa* from other isolates (S3 Fig).

**Fig 3:**
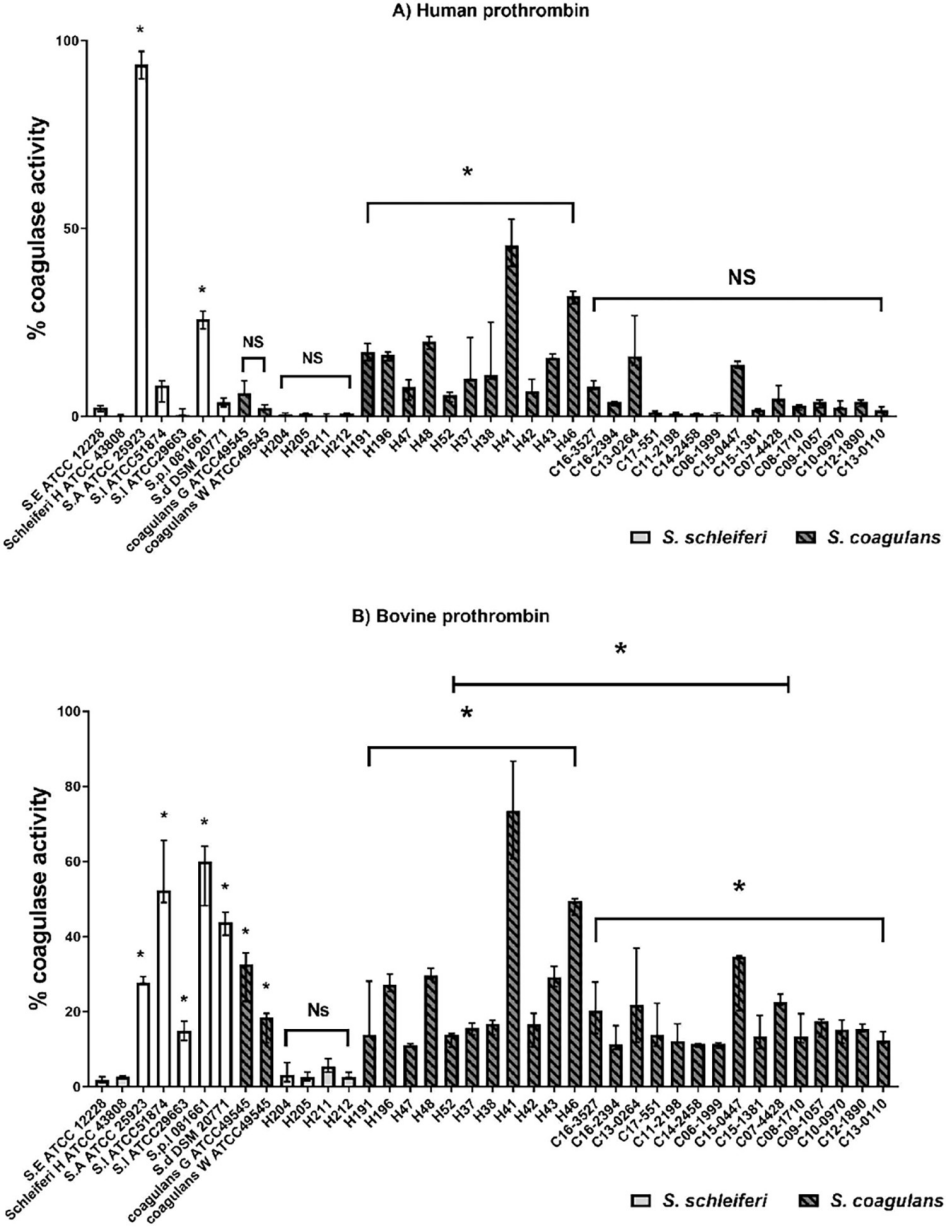
Coagulase activity. Coagulase activation of human (Panel A) and bovine (Panel B) prothrombin measured using a chromogenic assay. The amounts of coagulase activity in the bacterial supernatant of *S*. *coagulans* isolates from human (H) and canine(C) are indicated. Dashed columns are *S*. *coagulans*, light gray columns are *S*. *schleiferi* and white columns are control species. The values represent the medians from three independent experiments (*p<0.05, and NS p>0.05).

Coagulase activity was detected using human prothrombin substrate in supernatants from all *S*. *coagulans* and two *coa* PCR-positive *S*. *schleiferi* isolated from humans (H191 and H196) compared with coagulase-negative control strains (Fig 3A). A total of 10 out of the 13 isolates of *S*. *coagulans* and two *coa* PCR positive *S*. *schleiferi* of human origin activated human prothrombin. This compares to only 2 out of 17 isolates of *S*. *coagulans* strains from canine origin (C15-0447 p = 0.002 and C13-0264 p = 0.000) that significantly activated human prothrombin. All *S*. *coagulans* isolated from humans and canines and two *coa* PCR-positive *S*. *Schleiferi* isolated from humans (H191 and H196) were positive with bovine prothrombin. Bovine prothrombin was used in this study because, unlike canine prothrombin, it is commercially available and a comparison of the protein sequences of prothrombins showed that the amino acid identity between human and bovine prothrombin is 81.5%, whereas it is 89.3% between bovine and canine prothrombin and 84% between human and canine prothrombin.

Considered as groups, significant activation occurred with both bovine and human prothrombin with *S*. *coagulans* isolated from humans. *S*. *coagulans* strains from canine origin activated bovine prothrombin but not human prothrombin. As a comparison between coagulase-positive staphylococcal species to determine the biological significance of the coagulase activity, only *S*. *pseudintermedius* 081661 significantly activated human prothrombin (p = 0.000) and did not differ from *S*. *coagulans* strains of human origin (p = 0.748). All of the coagulase-positive staphylococcal species significantly activated bovine prothrombin p = 0.000 (*S*. *intermedius* ATCC 51874, *S*. *delphini* DSM 20771, *S*. *pseudintermedius* 081661, and *S*. *schleiferi* subsp. *coagulans* ATCC 49545 gray colony), p = 0.01 (*S*. *intermedius* ATCC 29663), and p = 0.022 (*S*. *schleiferi* subsp. *coagulans* ATCC 49545 white colony). The reactivity of *S*. *coagulans* isolates from canine and human origin with bovine prothrombin, analyzed as groups, were not significantly different from *S*. *schleiferi* subsp. *coagulans* ATCC 49545, *S*. *aureus* ATCC 25923, *S*. *intermedius* ATCC 29663 (p = 0.940) while significantly lower than the *S*. *pseudintermedius* 081661, *S*. *intermedius* ATCC 51874 (p = 0.000) and *S*. *delphini* DSM 20771 (p = 0.029).

### *clfB* gene amplification and detection of fibrinogen binding

**Fig 4:**
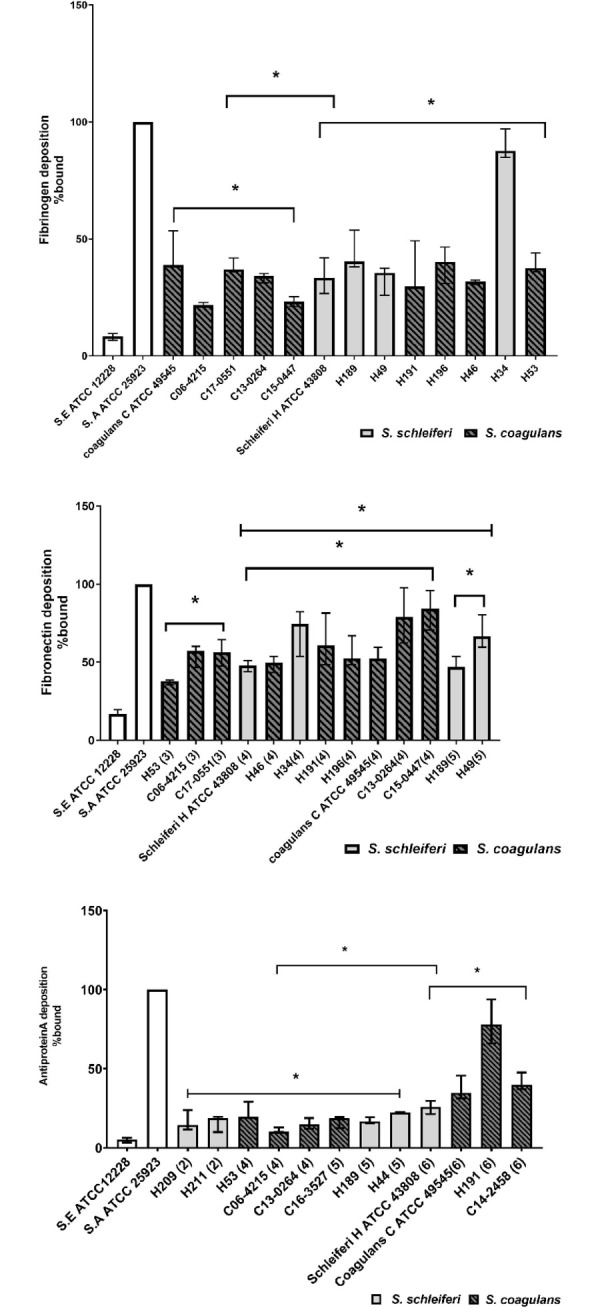
Ligand-binding assays. The amount of fibrinogen-binding protein (upper panel), fibronectin-binding protein (middle panel), and cell wall-associated protein A (lower panel) on the surface of *S*. *schleiferi*, and *S*. *coagulans* isolates of human (H) and canine (C) origin was measured by flow cytometry using FITC-conjugated fibrinogen HiLyte Fluor ^TM^ 488-conjugated fibronectin, and chicken anti-protein A antibody, respectively. Dashed columns are *S*. *coagulans*, light gray columns are *S*. *schleiferi* and white columns are control species. Numbers in parentheses indicate the number of fibronectin tandem repeats (middle panel), and the number of IgG binding domains (lower panel). The values represent the medians from three independent experiments (*p<0.05, and NS p>0.05).

The *clfB* gene of *S*. *schleiferi* encodes a protein that is 28% identical to clumping factor A (ClfA) of *S*. *aureus subsp*. *aureus* strain NCTC 8325 (AEK94092.1) and38% identical to clumping factor B (ClfB) of *S*. *aureus* st519 (WP_061742039.1). Only six strains (4 *S*. *schleiferi* and 2 *S*. *coagulans*) of human origin and one strain of *S*. *coagulans* of canine origin were positive for clumping factor genes (Table 2). *S*. *schleiferi* and *S*. *coagulans* of human (p = 0.004) and canine origin (p = 0.0283) were significantly higher in their amount of fibrinogen deposition than the negative control strain, *S*. *epidermidis* ATCC 12228 (Fig 4, upper panel).

**Table 2:** Comparison of phenotypic classification based on coagulase testing, and genotypic detection of *coa*, *clfb*, *spa*, *and fnB* genes.

Identification	Species level phenotypic classification based on urease and coagulase testing	sch- nuc (published Sasaki Primer)	Sch- nuc gene (This study)	*Coa* gene (Sch primer)	*Clfb* gene	*spa* IgG binding domains	Spa Type	FnB tandem repeats
ATCC 49545 T	coagulans	+ve	+ve	+	-	6	Spa-VI	4
ATCC 43808 T	schleiferi	Faint +ve	+ve	-	-	6	Spa-II	4
C 06–4215	coagulans	+ve	+ve	+	-	4	Spa-I	3
C 06–1999	coagulans	+ve	+ve	+	-	6	Spa-I	4
C 07–4428	coagulans	+ve	+ve	+	-	4	Spa-I	3
C 08–1710	coagulans	+ve	+ve	+	-	4	Spa-I	3
C 09–1057	coagulans	+ve	+ve	+	-	4	Spa-I	3
C 10–0970	coagulans	+ve	+ve	+	-	4	Spa-I	4
C 11–2198	coagulans	+ve	+ve	+	-	6	Spa-I	3
C 12–1890	coagulans	+ve	+ve	+	-	4	Spa-I	3
C 13–0264	coagulans	+ve	+ve	+	+	4	Spa-III	4
C 13–0110	coagulans	+ve	+ve	+	-	4	Spa-I	4
C 14–2579	coagulans	+ve	+ve	+	-	5	Spa-I	4
C14-2458	coagulans	+ve	+ve	+	-	6	Spa-I	4
C15-0447	coagulans	+ve	+ve	+	-	5	Spa-IV	4
C 15–1381	coagulans	+ve	+ve	+	-	4	Spa-I	3
C 16–3527	coagulans	+ve	+ve	+	-	5	Spa-IV	4
C 16–2394	coagulans	+ve	+ve	+	-	4	Spa-I	3
C 17–0551	coagulans	+ve	+ve	+	-	5	Spa-I	3
H 189	*Schleiferi*	Faint +ve	+ve	-	-	5	Spa-II	5
H 191	*Schleiferi*	+ve	+ve	+	+	6	Spa-I	4
H 192	*Schleiferi*	Faint +ve	+ve	-	-	5	Spa-II	5
H 194	*Schleiferi*	-ve	+ve	-	-	5	Spa-II	5
H 196	*Schleiferi*	+ve	+ve	+	+	6	Spa-I	4
H 204	*Schleiferi*	Faint +ve	+ve	-	-	5	Spa-II	4
H 205	*Schleiferi*	Faint +ve	+ve	-	-	5	Spa-II	4
H 209	*Schleiferi*	Faint +ve	+ve	-	-	2	Spa-II	4
H 211	*Schleiferi*	Faint +ve	+ve	-	-	2	Spa-II	4
H 212	*Schleiferi*	Faint +ve	+ve	-	-	6	Spa-II	4
H 214	*Schleiferi*	Faint +ve	+ve	-	-	5	Spa-II	4
H 34	*Schleiferi*	-ve	+ve	-	+	-	Spa-ND	4
H 35	*Schleiferi*	-ve	+ve	-	+	5	Spa-V	3
H 36	*Schleiferi*	Faint +ve	+ve	-	-	4	Spa-I	4
H 37	*Coagulans*	+ve	+ve	+	-	4	Spa-I	4
H 38	*Coagulans*	+ve	+ve	+	-	4	Spa-V	4
H 39	*Schleiferi*	Faint +ve	+ve	-	-	-	Spa- ND	4
H 40	*Coagulans*	+ve	+ve	+	-	5	Spa-I	4
H 41	*Coagulans*	+ve	+ve	+	-	4	Spa-V	4
H 42	*Coagulans*	+ve	+ve	+	-	4	Spa-V	4
H 43	*Coagulans*	+ve	+ve	+	-	4	Spa-V	4
H 44	*Schleiferi*	Faint +ve	+ve	-	-	5	Spa-IV	4
H 45	*Schleiferi*	-ve	+ve	-	-	-	Spa-ND	4
H 46	*Coagulans*	+ve	+ve	+	+	5	Spa-I	4
H 47	*Coagulans*	+ve	+ve	+	-	4	Spa-I	4
H 48	*Coagulans*	+ve	+ve	+	-	5	Spa-III	4
H 49	*Schleiferi*	Faint +ve	+ve	-	-	4	Spa-II	5
H 50	*Schleiferi*	Faint +ve	+ve	-	-	6	Spa-I	3
H 51	*Schleiferi*	Faint +ve	+ve	-	-	-	Spa-ND	5
H 52	*Coagulans*	+ve	+ve	+	-	4	Spa-IV	4
H 53	*Coagulans*	+ve	+ve	+	+	4	Spa-I	3
H 54	*Coagulans*	+ve	+ve	+	-	4	Spa-I	4
H 55	*Coagulans*	+ve	+ve	+	-	6	Spa-I	4

### Variation in the fibronectin-binding repeat region of *fnB* from *S*. *schleiferi* and *S*. *coagulans*

*fnB* was detected using a primer that amplified the repeat region and its DNA sequence showed variation in the length of C-terminal fibronectin-binding motifs into three distinct size classes. It was found that 52.9% of canine isolates produced three tandem repeats and 47% produced four tandem repeats. Among human isolates, 75.8% produced four tandem repeats, 93.3% were coagulase-positive and 61.1% were coagulase-negative. Among human isolates, 15.2% produced five tandem repeats and they were all coagulase-negative strains (Table 2).

The *fnB* gene of *S*. *schleiferi*, and *S*. *coagulans* encodes a protein with C-terminal fibronectin binding motifs that is 49% identical to *S*. *aureus* NCTC 8325 *fnB* (ABD31805.1). The variation in the number of fibronectin tandem repeats between the sequences of *S*. *schleiferi*, *S*. *coagulans* and *S*. *aureus* NCTC 8325 *fnB*, *S*. *schleiferi* 2317–03, *S*. *schleiferi* TSCC54and *S*. *schleiferi* 1360–13 (ABD31805.1, WP_050331312.1, BAS46387.1,and WP_050345785.1), respectively are shown in S4 Fig.

*S*. *schleiferi* and *S*. *coagulans* that have 3, 4 or 5 tandem repeats were significantly higher in their amount of fibronectin binding than *S*. *epidermedis* ATCC 12228 p = 0.0149, 0.002 and 0.025, respectively. Both *S*. *schleiferi*, and *S*. *coagulans* of human and canine origin bound lower amounts of fibronectin relative to *S*. *aureus* ATCC25923 except *S*. *coagulans* of canine origin (C13-0264 and C15-0447) that did not significantly differ from *S*. *aureus* (p = 0.068 for each). There was significant variation between *S*. *schleiferi and S*. *coagulans* that have 4 or 5 tandem repeats and *S*. *schleiferi* and *S*. *coagulans* that have 3 tandem repeats (p = 0.025) indicating that the number of fibronectin tandem repeats may affect fibronectin deposition on the cell wall of *S*. *schleiferi* and *S*. *coagulans* (Fig 4).

### *Spa* variation and detection of cell wall-associated protein A from *S*. *schleiferi* and *S*. *coagulans*

A *spa* PCR product was produced from all *S*. *schleiferi* and *S*. *coagulans* except 4 *S*. *schleiferi* isolates of human origin using the sch-spa primer. *Spa* gene length diverged into 4 different groups according to the numbers of IgG binding domains with 58.8%, 23.5%, and 17.6% of canine isolates and 33.3%, 33.3%, and 15.2% of human isolates producing 4, 5, or 6 IgG binding domains respectively. An additional 6.1% of *S*. *schleiferi* isolates of human origin contained 2 IgG binding domains. Interestingly, 58.8% and 60% of *S*. *coagulans* isolates of canine and human origin, respectively, had 4 IgG binding domains, while 44.4% of *S*. *schleiferi* isolates of human origin had 5 IgG binding domains. *Spa* of *S*. *schleiferi* and *S*. *coagulans* encoded a protein that is 60.2% identical to protein A of *S*. *aureus* Newman (BAF66327.1). The variation in the number of IgG binding domains between*S*. *schleiferi* and *S*. *coagulans* sequences and IgG binding domains of *S*. *aureus* Newman, *S*. *schleiferi* 2317–03, *S*. *schleiferi* TSCC54, and *S*. *schleiferi* 1360–13 (BAF66327.1, AKS72566.1, BAS44959.1 and AKS66049.1 respectively) are shown in S5 Fig.

*Spa* analysis of *S*. *schleiferi* and *S*. *coagulans* of canine and human origin showed 6 spa types using previously designed *spa* primers. The diversity of spa-types corresponds to the presence of the coagulase gene with 82.3% and 60% of *S*. *coagulans* isolates of canine and human origin respectively in spa type I and 26.7% of *S*. *coagulans* isolates of human origin in spa type V. While 55.6% of *S*. *schleiferi* of human origin and *S*. *schleiferi* subsp. *Schleiferi* ATCC 43808T of human origin are spa type II and none of the *S*. *coagulans isolates* of canine or human origin produce this spa type (Table 2).

## Discussion

Coagulase-negative staphylococci are considered commensal bacteria that lack important virulence factors such as those produced by *S*. *aureus*, and rarely contribute to clinical pathology [25]. In the case of *S*. *schleiferi*, however, this distinction is not as clear-cut as with other species of staphylococci. Both coagulase-positive *S*. *coagulans* and closely related, coagulase-negative *S*. *schleiferi* are associated with disease. *S*. *coagulans* are often distinguished from *S*. *schleiferi*, at least in part, by their positive tube coagulase test phenotype and urease activity [26]. We found, however, that some isolates identified as coagulase-negative using the standard tests have *coa* and are able to activate prothrombin. This draws into question the accuracy of the standard coagulase test in distinguishing *S*. *coagulans* from *S*. *schleiferi*. Staphylocoagulase genes have not been previously described for *S*. *coagulans* and in the current study, a gene corresponding to coagulase activity was identified in this species. Using human and bovine prothrombin as substrates in a coagulase chromogenic assay it was found that human-associated *S*. *coagulans* had the ability to activate human and bovine prothrombin. By contrast, most isolates from dogs had weak reactivity with human prothrombin but efficiently activated bovine prothrombin. *S*. *aureus* activation of human prothrombin was generally more than the amount seen with human-associated *S*. *coagulans* and *S*. *pseudintermedius*. In contrast, *S*. *aureus* and *S*. *coagulans* activated bovine prothrombin less than coagulase-positive *S*. *pseudintermedius*, *S*. *intermedius* and *S*. *delphini*. The differences in prothrombin activation may reflect the ability of *S*. *aureus* to coagulate human plasma more efficiently than bovine plasma, and the weak reactivity of *S*. *pseudintermedius* (originally identified as *S*. *intermedius*) with human prothrombin [27].

Variability in fibronectin adherence to *S*. *schleiferi* has been reported but its relation to the genetic heterogeneity of *S*. *schleiferi* and *S*. *coagulans* has not been described. Only *S*. *schleiferi* NCTC 12218 was previously tested, with a primer based on the *fnbA* of *S*. *aureus* 8325–4 [28, 29]. Analysis of *S*. *schleiferi* published genome sequences showed one gene with a variation in the C-terminal fibronectin-binding motifs. We found differences in the amount of fibronectin-binding protein between isolates related to that variation.

*S*. *schleiferi* has been reported to bind fibrinogen [30]. Flow cytometry analysis showed that all *S*. *schleiferi and S*. *coagulans* tested were able to bind fibrinogen, however, few isolates of *S*. *schleiferi* and *S*. *coagulans* contained the *clf* gene associated with fibrinogen binding. The most likely explanation is that it expresses one or more other genes responsible for fibrinogen binding. *fnB* was present in all *S*. *schleifer* and *S*. *coagulans* isolates. This gene has an N-terminal A domain that promotes binding to fibrinogen followed by tandemly repeated fibronectin-binding motifs [31].

Binding of *S*. *schleiferi* with protein A has been assessed using commercial agglutination kits [11] but the gene responsible for that binding has not been previously described. We found that the *S*. *schleiferi and S*. *coagulans spa* genes contain a variable number of IgG binding domains. *S*. *schleiferi* and *S*. *coagulans* that have more binding sites for IgG and fibronectin may adhere more strongly, be resistant to IgG, and be adapted to cause infection. Moreover, there are corresponding *spa* and coagulase genes within *S*. *schleiferi* and *S*. *coagulans* species with most of the *S*. *coagulans* having spa type-I and most *S*. *schleiferi* with spa type II.

Simultaneous detection of clumping factor and protein A has been reported to distinguish between the two *S*. *schleiferi* subspecies. However, we found both species may produce protein A but as stated above, are variable in their number of IgG binding domains whereas only a few isolates were negative for protein A production.

*S*. *coagulans* and *S*. *schleiferi* have been reported to have low genetic diversity [14] and targeting a portion of the *nuc* gene showed them to be phylogenetically indistinguishable [20]. A recent study concluded that *S*. *coagulans* and *S*. *schleiferi*, previously classified as subspecies of *S*. *schleiferi*, are distinct from each other, leading to their reclassification as *S*. *schleiferi* and *S*. *coagulans* [12].

In this study, we found a difference in *nuc* in human isolates negative for *coa* compared to *coa* positive isolates, suggesting that *nuc* genes differ between the two species. Using *nuc* primers based on currently available genomes may provide a rapid, accurate species-level identification of *S*. *schleiferi* and *S*. *coagulans* isolates of human and canine origin. Moreover, we identified a single nucleotide polymorphism in 16S rDNA that also distinguishes *S*. *schleiferi* and *S*. *coagulans*. These data suggest that *S*. *schleiferi*, and *S*. *coagulans* diverged and possibly became adapted to different hosts. A greater number of isolates would need to be examined to test this hypothesis.

## Materials and methods

### Bacterial isolates and control strains

A total of 50 *S*. *schleiferi* and *S*. *coagulans* isolates from human and canine sources were used in this study. Human isolates (n = 33) were collected in the United States for routine diagnostic procedures during a period between 2012 and 2016 and provided by clinical bacteriology laboratories for this study. The Director of the Human Research Protection program at the University of Tennessee determined that this study does not require Institutional Review Board review since it does not involve human subjects as defined by federal regulations. Canine isolates (n = 17) were obtained from the University of Tennessee College of Veterinary Medicine Clinical Bacteriology Laboratory during a period between 2006 and 2017. Each isolate was determined to be *S*. *schleiferi* or *S*. *coagulans* according to standard procedures which included colony morphology, a double zone of hemolysis on blood agar medium, positive Voges-Proskauer test, and negative maltose, trehalose, and lactose fermentation test results [10]. The species were distinguished from each other by urease and coagulase testing [11]. All canine isolates and 13 of the 33 human isolates were identified as *S*. *coagulans* and 20 human isolates were identified as *S*. *schleiferi*.

Control bacteria used for staphylocoagulase and ligand binding assays and PCR included the following coagulase-positive staphylococcal strains: *S*. *aureus* ATCC25923, *S*. *intermedius* ATCC 51874, *S*. *intermedius* ATCC 29663, *S*. *delphini* DSM 20771 T, *S*. *schleiferi* subsp. *coagulans* ATCC49545 T Gray colony canine origin, *S*. *schleiferi* subsp. *coagulans* ATCC49545 T white colony canine origin, and *S*. *pseudintermedius* strains 081661, E140, NA16, 063228, NA45 and E141. Coagulase-negative staphylococcal reference strains were *S*. *epidermidis* ATCC 12228, *S*. *sciuri* ATCC 29060, and *S*. *schleiferi* subsp. *schleiferi* ATCC 43808 T of human origin.

### DNA extraction and PCR amplification

Bacteria were grown on trypticase soy agar with 5% sheep blood overnight at 37°C. They were derived from a single colony, suspended in 5 ml of trypticase soy broth (TSB) (Becton, Dickinson and Co., Sparks, MD) and incubated on a rotary shaker at 225 rpm at 37°C. Bacteria were harvested from 1.8 ml of microbial culture and DNA was extracted using the DNeasy UltraClean Microbial Kit (Qiagen, Carlsbad, CA) according to the manufacturer’s protocol. Nine isolates were selected for *nuc* analysis. Human *S*. *Schleiferi* isolates H189, H192, and H205 were chosen because they produced a faint positive *nuc* PCR product, using a previously described *nuc* primer, and were negative for the coagulase gene. Human S. *Schleiferi* isolate H34 was included because it was negative by PCR for the *nuc* gene using a previously described *nuc* primer and the coagulase gene. Human *S*. *Schleiferi* isolates H191 and H196 were used because they were positive for the coagulase gene and identified as *S*. *Schleiferi* based on urease and coagulase testing. Canine *S*. *coagulans* isolates C08-1710, C16-3527, and C17-0551 were chosen because they were positive for the coagulase gene and identified as *S*. *coagulans* based on urease and coagulase testing.

PCR was performed using primers (Table 1) previously described for *nuc* [20], the 16S rRNA gene [32], *fnbA* [28], *spa* forward primer spaT3 [33], reverse primer 1517R [33], and *coa* [19]. Additional PCR primers were designed using an online tool (Integrated DNA Technologies, Coralville, IA) based on a multiple sequence alignment of *S*. *schleiferi* isolates 5909–02, 1360–13, 2142–05, 2317–03 and TSCC54 using genomic sequence data from the GenBank database (CP009676, CP009470, CP009762, CP010309 and AP014944, respectively) [34, 35]. They included forward and reverse primers for Sch-nuc, Sch-coa, sch-clfB, fnbB, and sch-spa. The reaction mixtures consisted of a 25 μl total volume containing 2.5 μl of genomic DNA, 20 pmol of each primer (1 μl), 12.5 μl of rTaq polymerase enzyme, and 9 μl of nuclease-free water. The following cycling conditions were performed for *nuc*, *spa*, *clfB* and *fnbB*: initial denaturation at 95°C for 1.5 min, annealing at 50°C for 30s, extension at 72°C for 2.5min (30 cycles), and a final extension at 72°C for 5min. *coa* amplification conditions consisted of an initial denaturation (95°C for 1.5min), annealing at 55°C for 30s, extension at 68°C for 2 min (30 cycles), and a final extension at 68°C for 5min. PCR products were resolved and visualized in 1.4% agarose gels. The size of PCR products was confirmed using capillary electrophoresis (QIAxcel Advanced System).

### Sequence analysis

PCR products were enzymatically treated to destroy single-stranded DNA (ExoSap-IT, USB Corp., Cleveland, OH) and were sequenced at the University of Tennessee, Knoxville Genomics Core Facility. The sequences of the genes were aligned and compared (Geneious, Biomatters, Auckland, New Zealand). Protein sequences were predicted from each DNA sequence using an online tool (Expasy translate, http://web.expasy.org/translate/)and phylogenetic trees were generated using Phylogeny.fr [36].

### Chromogenic staphylocoagulase assay

A chromogenic assay was used to measure the activity of staphylocoagulase as previously described [19] to determine if there were consistent differences between the *S*. *schleiferi* and *S*. *coagulans* or the source of isolation. Single colonies of bacterial isolates were cultured overnight in 2 ml of TSB at 37°C. Bacteria were centrifuged at 12000 X g for 2 min then supernatants were concentrated using Amicon Ultra-0.5 centrifugal 500 μl (Millipore) 30KDa filters. Testing was performed in flat-bottom microtiter plates. The molar concentrations of prothrombin were calculated using molecular mass estimates of 72,000 daltons for human and bovine prothrombin. A 20 μl aliquot of concentrated supernatant was mixed with 1x10^-16^ M of human or bovine prothrombin and incubated for 30 min at 37°C. Thrombin tripeptide substrate H-D-Phe-Pip-Arg-pNA (Molecular innovations, Novi, MI) was added to a final concentration of 1 mM in a total reaction buffer volume of 100 μl PBS per well. After an initial reading, the reaction was allowed to proceed by incubating in the dark for 1, 4, and 8h at 37°C. Absorbance was measured at 405 nm. Coagulase-positive and coagulase-negative staphylococcal type strains were included with each batch as quality controls. Prothrombin activation by *S*. *schleiferi* and *S*. *coagulans* isolates was compared with that of *S*. *aureus* ATCC 25923, which activates prothrombin and coagulase-negative *S*. *epidermidis* ATCC 12228 [37, 38]. The change in absorbance over the time of incubation (dA/dt*100) was plotted and interpreted as the rate of substrate hydrolysis that reflects the enzymatic function of coagulase [39].

### Fibrinogen and fibronectin deposition and anti-protein A reactivity

Bacteria derived from a single colony were suspended in 5 ml of TSB and incubated in a rotary shaker at 225 rpm at 37°C. They were harvested from 100μl of microbial culture, then washed and standardized to a 600 nm optical density of 0.5 and incubated with either no conjugate (NC, negative control) or 5μg/ml of chicken anti-protein A fluorescein isothiocyanate (FITC) conjugate (Gallus Immunotech, Cary, NC), 20 μg/ml human fibrinogen FITC conjugate (Zedira GmbH, Darmstadt, Germany) or 10 μg/ml bovine fibronectin HiLyte Fluor TM 488 conjugate (Cytoskeleton Inc., Denver, CO) for 30 min at 37°C in the dark. Excess conjugate was removed by washing. For each sample, the amount of binding was determined by measuring the fluorescence intensity of 10,000 bacteria using a flow cytometer (Attune acoustic focusing cytometer, Applied Biosystems, Foster City, CA) at excitation/emission wavelengths of 488/519 nm. Bacteria were gated based on forward and side scatter profiles. Binding of fibrinogen, fibronectin, and anti-protein A to *S*. *schleiferi* and *S*. *coagulans* isolates was compared with that of *S*. *aureus* ATCC25923, which is known to bind these ligands and display protein A on its surface [37], and with that of *S*. *epidermidis* ATCC 12228, which binds fibrinogen and fibronectin poorly and does not express protein A [38].

The percent of bound ligand was normalized to binding by *S*. *aureus* ATCC 25923 calculated as: B/B0*100 where B is the mean fluorescence intensity of a *S*. *schleiferi* or *S*. *coagulans* sample and B0 is the mean fluorescence intensity of *S*. *aureus* ATCC 25923. The fold change between the higher and lower binder of *S*. *schleiferi* and *S*. *coagulans* was measured by the following equation:(B—A)/A where the lowest value is A and highest value is B.

### Statistical analysis

ANOVAs and multiple comparisons using Turkey’s and Dunnett’s post hoc tests were performed to determine if there was a significant difference in prothrombin activation among bacterial isolates and to determine if there was a significant difference in the amount of surface-bound protein A, fibrinogen-binding protein and fibronectin-binding protein. Each experiment was repeated at least three times and all post hoc tests used a Bonferroni adjustment considering p-value of <0.05 as significant. All analyses were conducted using SPSS Statistics for Windows, Version 24.0 (IBM Corp, Armonk, NY). All graphs were prepared using the GraphPad Prism software (Version 7, GraphPad Software Inc.).

## Supporting information

S1 Fig *nuc* gene multiple sequence alignment. Clustal W alignment of *nuc* gene sequence of *S*. *schleiferi* (sequence profile I) and *S*. *coagulans* (sequence profile II) of human (H) and canine (C) origin. The 16 nucleotide difference between the two sequence profiles is highlighted by gray shading.
(TIF)

S2 Fig 16S rRNA multiple sequence alignment. Clustal W alignment of 16S rRNA gene sequence of *S*. *schleferi* (sequence profile I) and *S*. *coagulans* (sequence profile II) of human (H) and canine (C) origin. The single nucleotide difference between the two sequence profiles is highlighted by gray shading.
(TIF)

S3 Fig Coagulase binding domains. Clustal W alignment of prothrombin binding domains D1, D2 (Panel A) and the fibrinogen binding region (Panel B) of coagulase protein sequences among 22 species of the genus *Staphylococcus* including 15 *S*. *coagulans* of human (H) and canine (C) origin. The following staphylocoagulase protein sequences were obtained from the GenBank database (accession numbers): *S*. *aureus subsp*. *aureus* strain Newman (WP_000744074), *S*. *aureus* strain 6850 (WP_020977090.1) *S*. *intermedius* ATCC 29663(WP_019169028.1), *S*. *schleiferi* 1360–13 (WP_050345467.1), *S*. *schleiferi*2317-03 (WP_050330609.1), *S*. *delphini* 8086 (WP_019166910.1), and *S*. *pseudintermedius* 081661 (WP_037544060.1). The annotation label indicates the prothrombin binding domains and fibrinogen repeat region (Coa-Ro, RI, RII, RIII, and RIV). White, red, gray, and blue shading indicate 100%, 80–100%, 60–80%, and less than 60% similarity between sequences, respectively.
(PDF)

S4 Fig Fibronectin binding protein. A) Binding site of FnbpB primers used in this study showed variation in the length of C-terminal fibronectin binding motifs among the *S*. *schleiferi* published sequences B) CLUSTALW alignment of the amino acid sequences of the fibronectin tandem repeats of FnB protein among six *S*. *schleiferi* and *S*. *coagulans* isolates of human (H) and canine (C) origin in comparison with *S*. *aureus* NCTC 8325 FnB. The annotation label indicates the fibronectin repeat region (1–5). Numbers between braces indicate the number of fibronectin tandem repeats. Gray shading indicates 80–100% similarity between sequences.
(PDF)

S5 Fig Protein A binding domains. A) Binding site of *spa* primers used in this study showed variation in the length of IgG binding domains of protein A among the *S*. *schleiferi* published sequences. B) CLUSTALW alignment of the amino acid sequences of the IgG binding domains of protein A among 7 *S*. *schleiferi* and *S*. *coagulans* isolates of human (H) and canine (C) origin in comparison with *S*. *aureus* Newman protein A. The annotation label indicates the number of IgG binding domains (I-V). Gray shading indicates 80–100% similarity between sequences.
(PDF)
