# Supplementary figures and images for: Distinguishing characteristics of Staphylococcus schleiferi and Staphylococcus coagulans of human and canine origin
Source: PLoS One. 2024 Feb 8;19(2):e0296850. doi: 10.1371/journal.pone.0296850 (PMC10852249; doi:10.1371/journal.pone.0296850)

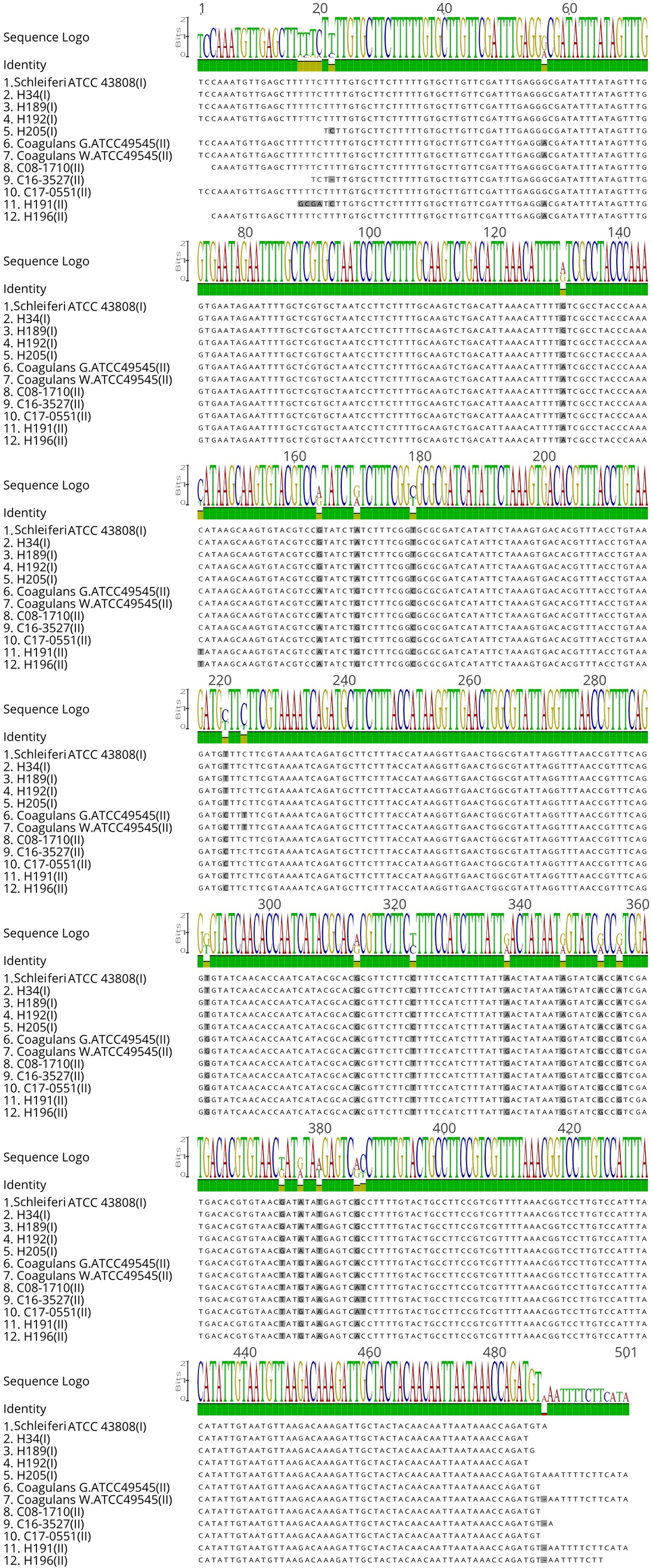

Supplement: S1 Fig — Clustal W alignment of nuc gene sequence of S. schleiferi (sequence profile I) and S. coagulans (sequence profile II) of human (H) and canine (C) origin. The 16 nucleotide difference between the two sequence profiles is highlighted by gray shading. (TIF) [file pone.0296850.s001.tif]

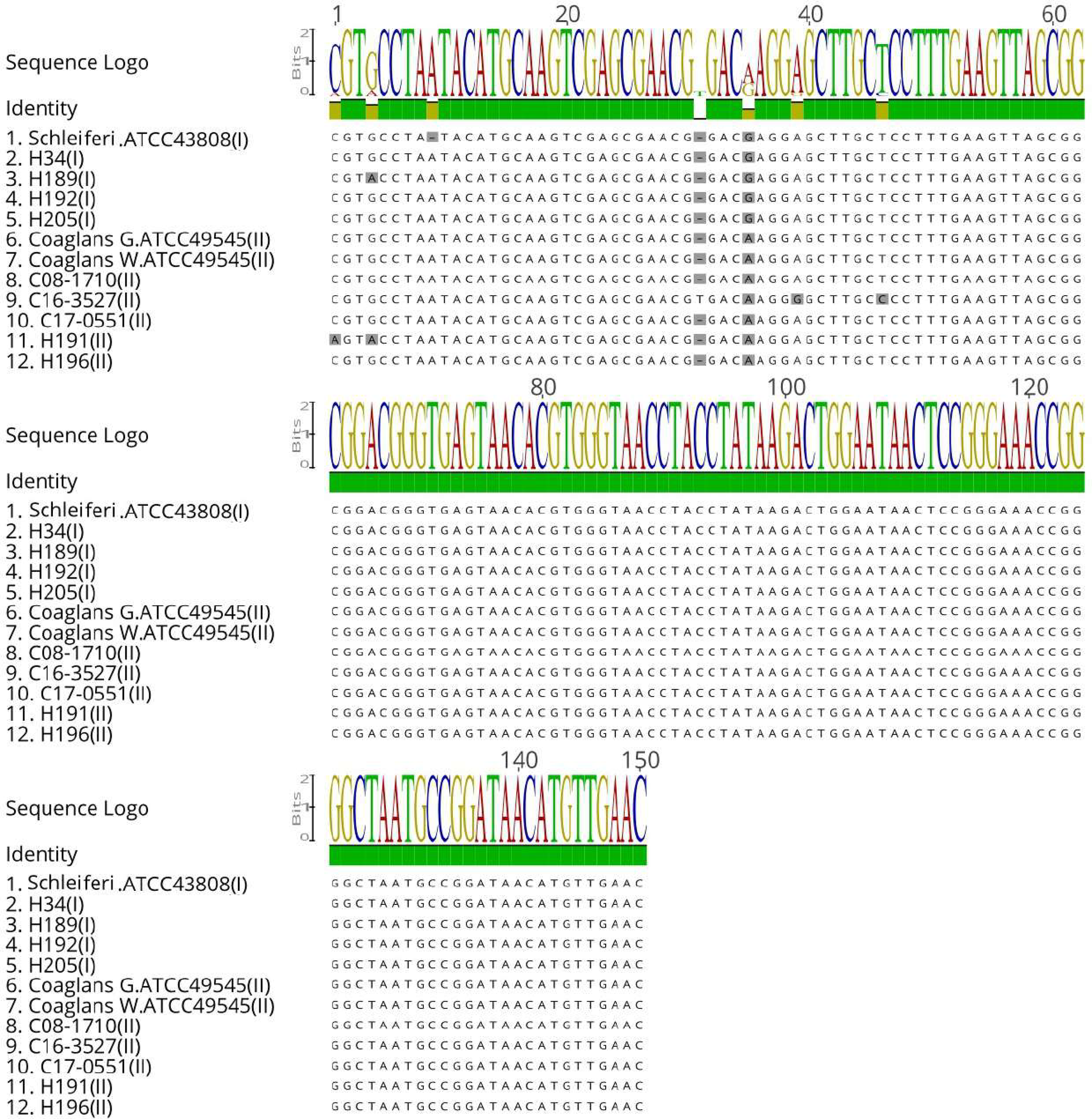

Supplement: S2 Fig — Clustal W alignment of 16S rRNA gene sequence of S. schleferi (sequence profile I) and S. coagulans (sequence profile II) of human (H) and canine (C) origin. The single nucleotide difference between the two sequence profiles is highlighted by gray shading. (TIF) [file pone.0296850.s002.tif]

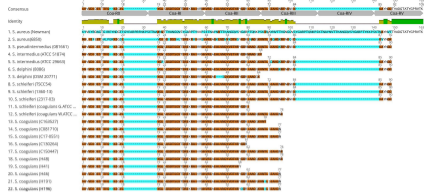

Supplement: S3 Fig — Clustal W alignment of prothrombin binding domains D1, D2 (Panel A) and the fibrinogen binding region (Panel B) of coagulase protein sequences among 22 species of the genus Staphylococcus including 15 S. coagulans of human (H) and canine (C) origin. The following staphylocoagulase protein sequences were obtained from the GenBank database (accession numbers): S. aureus subsp. aureus strain Newman (WP_000744074), S. aureus strain 6850 (WP_020977090.1) S. intermedius ATCC 29663(WP_019169028.1), S. schleiferi 1360–13 (WP_050345467.1), S. schleiferi2317-03 (WP_050330609.1), S. delphini 8086 (WP_019166910.1), and S. pseudintermedius 081661 (WP_037544060.1). The annotation label indicates the prothrombin binding domains and fibrinogen repeat region (Coa-Ro, RI, RII, RIII, and RIV). White, red, gray, and blue shading indicate 100%, 80–100%, 60–80%, and less than 60% similarity between sequences, respectively. (PDF) [file pone.0296850.s003.pdf]

# Spa gene

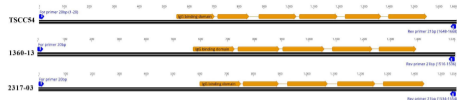

A

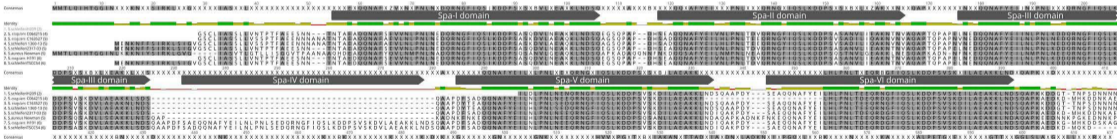

B

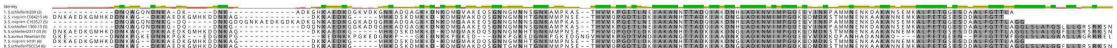

Supplement: S5 Fig — A) Binding site of spa primers used in this study showed variation in the length of IgG binding domains of protein A among the S. schleiferi published sequences. B) CLUSTALW alignment of the amino acid sequences of the IgG binding domains of protein A among 7 S. schleiferi and S. coagulans isolates of human (H) and canine (C) origin in comparison with S. aureus Newman protein A. The annotation label indicates the number of IgG binding domains (I-V). Gray shading indicates 80–100% similarity between sequences. (PDF) [file pone.0296850.s005.pdf]
