# Supplementary material for: Distinguishing characteristics of Staphylococcus schleiferi and Staphylococcus coagulans of human and canine origin
Source: PLoS One. 2024 Feb 8;19(2):e0296850. doi: 10.1371/journal.pone.0296850 (PMC10852249; doi:10.1371/journal.pone.0296850)
Supplement: S4 Fig — A) Binding site of FnbpB primers used in this study showed variation in the length of C-terminal fibronectin binding motifs among the S. schleiferi published sequences B) CLUSTALW alignment of the amino acid sequences of the fibronectin tandem repeats of FnB protein among six S. schleiferi and S. coagulans isolates of human (H) and canine (C) origin in comparison with S. aureus NCTC 8325 FnB. The annotation label indicates the fibronectin repeat region (1–5). Numbers between braces indicate the number of fibronectin tandem repeats. Gray shading indicates 80–100% similarity between sequences. (PDF) [file pone.0296850.s004.pdf]

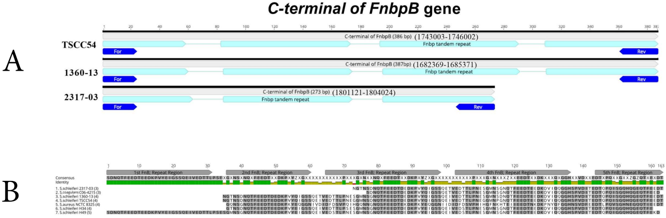
C-terminal of FnbpB gene
A
TSCC54
C-terminal of FnbpB (386 bp) (1743003-1746002)
Fnbp tandem repeat
For
Rev
1360-13
C-terminal of FnbpB (387bp) (1682369-1685371)
Fnbp tandem repeat
For
Rev
2317-03
C-terminal of FnbpB (273 bp) (1801121-1804024)
Fnbp tandem repeat
For
Rev
B
1st FnB; Repeat Region
2nd FnB; Repeat Region
3rd FnB; Repeat Region
4th FnB; Repeat Region
5th FnB; Repeat Region
Consensus
Identity
1. S.schleiferi 2317-03 (3)
2. S.coagulans C06-4215 (3)
3. S.schleiferi 1360-13 (4)
4. S.schleiferi TSCC54 (4)
5. S.aureus NCTC 8325 (4)
6. S.schleiferi H34 (4)
7. S.schleiferi H49 (5)
